# Diabetic Cardiomyopathy—From Basics through Diagnosis to Treatment

**DOI:** 10.3390/biomedicines12040765

**Published:** 2024-03-29

**Authors:** Ewa Radzioch, Bartłomiej Dąbek, Marta Balcerczyk-Lis, Weronika Frąk, Piotr Fularski, Ewelina Młynarska, Jacek Rysz, Beata Franczyk

**Affiliations:** 1 Department of Nephrocardiology, Medical Univeristy of Lodz, ul. Zeromskiego 113, 90-549 Lodz, Poland; 2 Department of Nephrology, Hypertension and Family Medicine, Medical University of Lodz, ul. Zeromskiego 113, 90-549 Lodz, Poland

**Keywords:** diabetic cardiomyopathy, diabetes mellitus, molecular mechanisms, risk factors, diagnosis, therapy

## Abstract

Diabetic cardiomyopathy (DCM) is the development of myocardial dysfunction in patients with diabetes despite the absence of comorbidities such as hypertension, atherosclerosis or valvular defect. The cardiovascular complications of poorly controlled diabetes are very well illustrated by the U.K. Prospective Diabetes Study (UKPDS), which showed a clear association between increasing levels of glycated hemoglobin and the development of heart failure (HF). The incidence of HF in patients with diabetes is projected to increase significantly, which is why its proper diagnosis and treatment is so important. Providing appropriate therapy focusing on antidiabetic and hypolipemic treatment with the consideration of pharmacotherapy for heart failure reduces the risk of CMD and reduces the incidence of cardiovascular complications. Health-promoting changes made by patients such as a low-carbohydrate diet, regular exercise and weight reduction also appear to be important in achieving appropriate outcomes. New hope for the development of therapies for DCM is offered by novel methods using stem cells and miRNA, which, however, require more thorough research to confirm their efficacy.

## 1. Introduction

**Figure 1:**
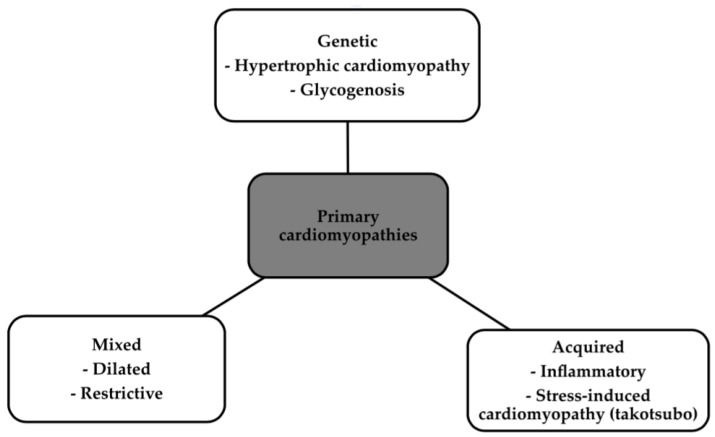
Classification of primary cardiomyopathies with some examples [3].

**Figure 2:**
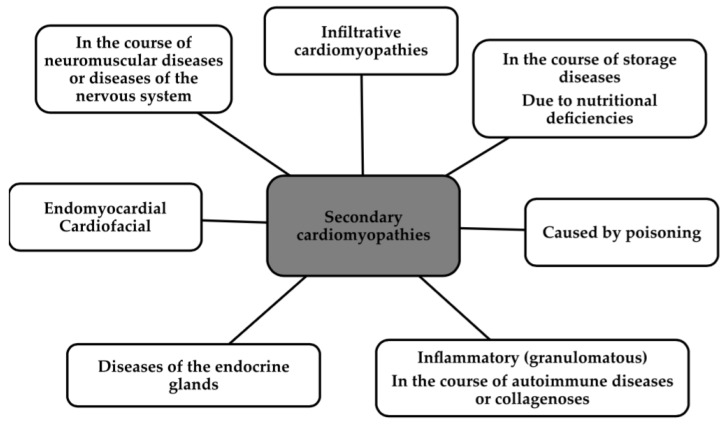
Classification of secondary cardiomyopathies [3].

Over the years, the definition of cardiomyopathy has undergone numerous changes [1]. Cardiomyopathies are primarily a heterogeneous set of pathologies involving both functional and structural changes to the heart [2]. The classic division divides it into two major branches, namely primary, in which the disease is localized in the heart, and secondary, which is a component of other diseases [1,2]. Figure 1 and Figure 2 show the classification of the primary cardiomyopathies with some examples and the secondary ones according to the American Heart Association, to show how widespread the problem is [3].

In this publication, we will focus primarily on diabetic cardiomyopathy (DCM), i.e., secondary cardiomyopathy in the course of endocrine gland diseases, which is caused by diabetes and leads to heart failure (HF) [4]. DCM has been defined by the American College of Cardiology Foundation, American College of Cardiology and the European Society of Cardiology in collaboration with the European Association for the Study of Diabetes as myocardial dysfunction without concomitant coronary atherosclerosis and elevated blood pressure [5,6]. Therefore, all patients with cardiac dysfunction without significant coronary artery disease, without valvular disease and hypertension could be classified as patients with DCM [7]. Defining this disease correctly is quite a challenge, in view of the fact that most patients with type 2 diabetes mellitus (T2DM) have cardiovascular disease (CVD). Thus, some authors define DCM as cardiac disorders that are not explainable by other cardiac diseases [8]. The incidence of diabetic cardiomyopathy increases in proportion to the onset of diabetes in the population [9]. Despite the growing clinical possibilities, the process of DCM formation is still a kind of unsolved disease entity for us [10]. In this publication, we will try to introduce the disease, its diagnosis and the latest treatment options.

## 2. Pathophysiology

The pathophysiology of DCM is being studied, discovered and explained all the time [11]. There are a great many theories that probably influence the appearance of this disease [12].

**Figure 3:**
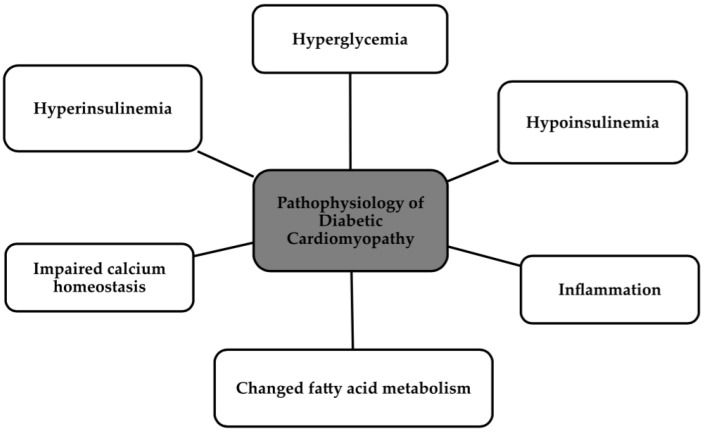
Pathophysiology of DCM [16].

DCM was confirmed as a new clinical entity in the early 1970s, after postmortem studies were carried out on four patients who had both type 2 diabetes and HF. The study in animal models with both T1DM and T2DM detected specific changes in cardiomyocyte proteins, which led to the idea that abnormal cardiomyocyte contractile function could be added to the definition [4,13]. DCM is associated with left ventricular hypertrophy along with either systolic dysfunction or diastolic dysfunction, which can be in the form of HF with preserved ejection fraction (HFpEF) and fatty acid (FA) accumulation in cardiomyocytes [11,14]. DCM, caused by the presence of diabetes in patients, impairs the myocardium in several different respects. Microvascular abnormalities occur, metabolic dysfunctions develop, in addition to autonomic abnormalities and inadequate immune responses [11]. The two main metabolic abnormalities are hyperglycemia and insulin resistance [12]. Others include metabolic abnormalities, inflammation, endothelial damage, oxidative stress, lipotoxicity, abnormal function of the RAAS system, and abnormal calcium homeostasis [11,12,15,16,17]. The pathophysiology in the form of several mechanisms is shown in the Figure 3 below [16].

These phenomena are responsible for myocyte necrosis, interstitial myocardial damage, apoptosis or mitochondrial damage. Changes in calcium homeostasis, which is one of the mechanisms, are caused by oxidative stress. This causes a decrease in coronary nitric oxide (NO) synthase, which causes an increase in intracellular calcium, which consequently reduces calcium reuptake in the cytoplasm and increases calcium leakage from the cytoplasm. This entire mechanism leads to calcium accumulation during cardiac diastole, decreases the muscle’s relaxation capabilities and increases its stiffness, resulting in abnormal cardiac contraction [12,16]. Diabetes induces a chronic inflammatory state in the body, with increased levels of the nucleotide-binding oligomerization domain-like receptor family, pyrin domain-containing 3 (NLRP3) inflammasome. NLRPR3 is thought to be responsible for the development of DCM. NLRP3 is activated by conditions such as hyperglycemia, high FFA levels or abnormal metabolic insulin signaling [7]. 

Excessively high blood glucose levels generate the production of interleukins (ILs), IL-1β, IL-6, IL-18, TNF-α and TGF-β1, monocyte chemoattractant protein 1, tumor necrosis factor or factor κB causing myocardial cell degradation [16,17]. In addition, the above-mentioned ILs, such as IL-18, IL-1β and NF-kB, when activated by NLRP3, cause local tissue inflammation [7].

Hyperglycemia additionally causes the activation of protein kinase C pathways, resulting in free radical production and myocardial necrosis. Dystrophic calcification, myocardial fibrosis and left ventricular hypertrophy occur [9,18]. Hearts in humans and animal models with T1DM and T2DM showed increased FA uptake and oxidation. Lipotoxicity may adversely affect cardiomyocyte function. This is most likely due not only to the increased concentration of triglycerides (TG) and FA in the blood, but also to the increased availability of intermediate forms of fats such as oxidized phospholipids and ceramides [17]. The formation of intermediates, i.e., diacylglycerol, has a NO synthase-mediated effect on reducing muscle cell expansion, affecting the microcirculatory system at the same time [15]. As the frequency of FA oxidation increases in the heart, there is an increased consumption of oxygen by cardiac muscle cells, resulting in pathological cardiac remodeling, cardiac steatosis and decreased performance [17,18]. It has also been shown that the metabolic reserve of the heart is impaired in diabetes and this is due to the response of cardiomyocytes to insulin, stress and the changing amount of available FA. Consequently, there is reduced glucose utilization and increased fatty acid oxidation [19]. In patients with insulin resistance and hyperinsulinemia, with co-morbid obesity, there is an increase in both FA and serum glucose. A systemic inflammatory response occurs, resulting in the generation of free radicals, and in addition, the heart uses less favorable energy sources, resulting in the formation of less ATP. Excessively high blood insulin concentrations, in turn, activate a pathway that induces remodeling and hypertrophy of cardiomyocytes [15].

FA are carried across mitochondrial membranes, resulting in mitochondrial dysfunction and fusion. Studies in mouse models have shown that mitochondrial fragmentation and decreased mitochondrial ordering results in myocardial dysfunction [20]. The myocardial metabolism is a promising target for upcoming research and will make it possible in the near future to answer the question of how diabetic substrates could be prevented from developing multiple cardiac dysfunctions [21]. It is worth noting that cardiomyopathy in DM is a multifactorial disease that often includes myocardial fibrosis as well as LV dysfunction at the same time [22]. Insulin resistance and altered glucose metabolism affect excessive sympathetic activation, causing myocardial fibrosis. This leads to sympathetic denervation and abnormal β-adrenergic signaling. This results in decreased myocardial contractility and impaired overall myocardial kinetics [15].

We have two possible pathways in which myocardial fibrosis can occur, which simultaneously generate its division into replacement fibrosis and interstitial fibrosis. Replacement fibrosis is caused by myocardial infarction and is treated as a response to the repair of damaged tissue. Interstitial fibrosis, on the other hand, is caused by various pathologies such as hypertension, HF or just diabetes [20].

The U.K. Prospective Diabetes Study (UKPDS), which included more than 4500 patients, proved that an increase in glycated hemoglobin clearly correlated with an increased risk of HF over the next 10 years [14]. DCM can result from a variety of pathophysiological processes occurring in both type 1 diabetes mellitus (T1DM) and T2DM. In T1DM, autoimmune processes are partly responsible for the cardiac disorders that occur, which in T2DM are caused by coexisting obesity, hyperglycemia, hyperinsulinemia, or dyslipidemia [23]. Cardiac dysfunction affects both types of diabetes, but the pathophysiology remains incompletely uncovered. Type 1 diabetes is thought to involve mitochondrial dysfunction, while type 2 diabetes involves abnormal mitochondrial respiration. Recent studies in animal models assessing mitochondrial dysfunction show abnormalities in both number, structure and function [24]. 

A 2023 publication showed that mitochondria in people with type 2 diabetes, relative to healthy individuals, have a significantly shorter average length and are significantly smaller than in healthy individuals. Reduced fusion and mitochondrial fission have been observed in patients with DCM and in animal models [25].

**Figure 4:**
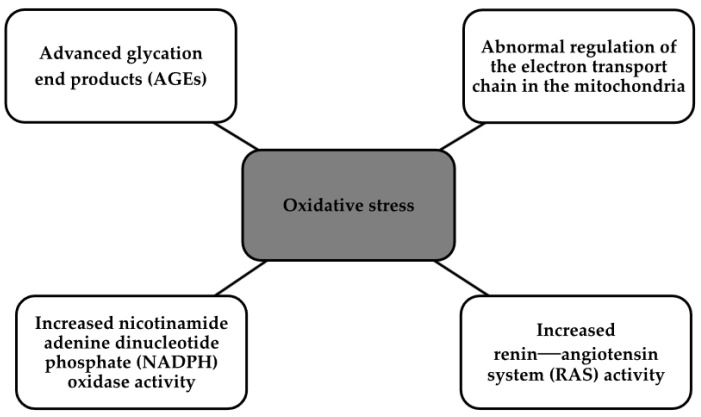
Oxidative stress [7].

The mechanisms by which diabetes increases oxidative stress have been discovered. These are shown in the Figure 4 below [7].

It has also been proven that oxidative stress and inflammation increase the production of free radicals, thereby increasing cardiac dysfunction and remodeling. Fibrosis and myocardial hypertrophy occur. The study also uncovered the role of hydrogen sulfide H2S, which caused mitochondrial damage, increased free radicals and activated NLRP3, resulting in the exacerbation of DCM in test animals [7,26].

## 3. Epidemiology

Epidemiological data highlight a significant correlation between DM and HF [27].

Research was carried out to examine the occurrence and predictive significance of diabetic cardiomyopathy (DCM) among community-dwelling patients. One aspect under investigation was the five-year occurrence of heart failure (HF) among individuals with DCM. Findings indicated that, in comparison to the euglycemia group, irrespective of the severity of the condition, DCM was notably linked to an increased risk of developing heart failure [28]. Findings from the Framingham study revealed a 2.4-fold rise in HF incidence among diabetic men and a 5-fold increase in diabetic women. Moreover, HF emerges as a more frequent initial manifestation of CVD in individuals with diabetes mellitus (DM) compared to myocardial infarction [27,29]. The frequency of DM among patients with HF varies between 10 and 30%, with rates escalating to 40% among individuals hospitalized for acute conditions. Projections suggest a significant surge in these figures in the coming decades, attributed to the expanding elderly demographic. DM serves as an autonomous predictor of cardiovascular morbidity and mortality in individuals experiencing chronic symptomatic HF, regardless of whether they have HFpEF or reduced ejection fraction (HFrEF). This underscores its consistent diagnostic significance across both categories [30]. The association between DM and HF works both ways: the presence of DM worsens outcomes for HF patients, while individuals with DM are at a heightened risk of developing HF. Not only is the mere presence of DM relevant, but the level of glycemic control also correlates with the risk of HF. Research indicates that with every 1% rise in hemoglobin A1C (HbA1C), there is an 8% increase in HF risk. Given the strong epidemiological link between HF and DM, HF should now be recognized as a crucial cardiovascular outcome for evaluating the effectiveness of new glucose-lowering therapies [31]. Research has demonstrated that South Asians, in comparison to other ethnicities, face an elevated risk of developing T2DM and, consequently, are more susceptible to developing DCM. It is suspected that they might possess a metabolically unfavorable phenotype characterized by a relatively high total body fat percentage. Additionally, their metabolic response to excess fat mass may be more pronounced, as suggested by heightened insulin resistance levels at comparable levels of adiposity [32]. The pathophysiology and clinical characteristics of DCM can vary between different types of DM. For instance, systolic dysfunction is more frequently observed in T1DM compared to T2DM, although diastolic dysfunction can occur in both groups [20]. However, there remains a scarcity of data concerning the relationship between T1DM and the risk of HF. To address this gap, a meta-analysis was conducted in 2023, encompassing 61,885 T1DM patients, a control group comprising 4,599,213 non-diabetic individuals, and 248,021 patients with T2DM. The analysis investigated the association between diabetes type and the likelihood of future HF events among these cohorts. The results indicated the highest correlation in T2DM patients, followed by T1DM, with the lowest correlation observed in the control group. Specifically, T1DM patients exhibited a threefold higher risk of HF compared to the control group [33]. There is no denying that DM raises the occurrence of diastolic dysfunction, with rates reaching 50% in individuals aged over 65 years. Nishi et al. conducted stress echocardiograms on 161 DM patients to assess subclinical heart failure rates, revealing higher rates of diastolic dysfunction during stress compared to rest (57% versus 45%, respectively) [34].

## 4. Risk Factors

**Figure 5:**
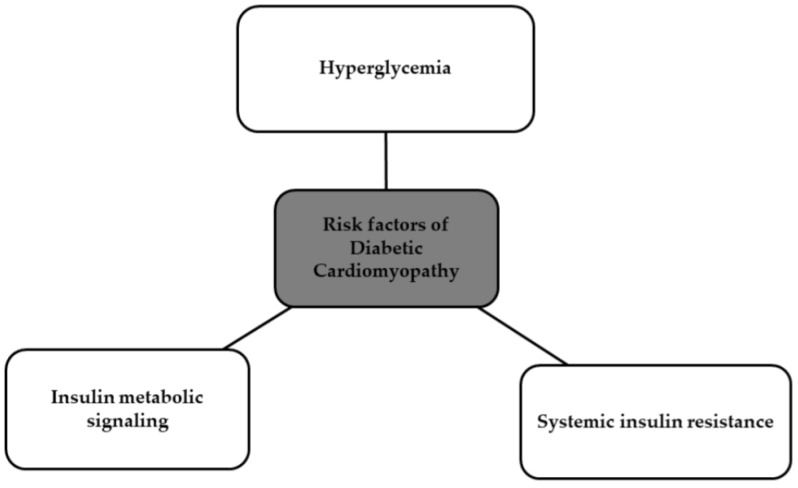
Risk factors of DCM [35,36].

The major risk factors that are the most closely correlated with DCM are shown in the Figure 5 below [35,36].

In a prospective nationwide survey involving HF patients, 1811 individuals with pre-existing DM and 2182 without, with blood glucose levels ranging between 110–140, 140–200, and ≥200 mg/dL were associated with a 9%, 16%, and 53% heightened risk of mortality, respectively, compared to those with admission blood glucose levels below 110 mg/dL among patients without pre-existing DM. A direct correlation was observed between blood glucose levels and long-term mortality in HF, even among patients without a clinical diagnosis of DM. Conversely, elevated mortality risk was only evident in diabetic individuals with blood glucose levels exceeding 200 mg/dL [36,37]. The current rise in the consumption of refined carbohydrates in the diet, particularly fructose, might also influence the development of DCM [36]. A more recent study, conducted in 2023, involved a 16-week randomized, controlled pilot trial comparing the impacts of a low-carbohydrate diet against usual care in individuals with DCM. Seventeen participants were enrolled (thirteen finished), and changes in variables such as body weight, systolic blood pressure, and thirst distress were assessed. Among the parameters examined, it was found that the low-carbohydrate diet led to a significant reduction in body weight among patients with DCM. While the findings are promising, conducting the study over a longer duration and on a larger cohort of subjects is necessary to establish more robust conclusions [38].

There are also indications that consuming a diet rich in fats and processed carbohydrates leads to disrupted cardiac insulin metabolic signaling, inflammation, oxidative stress, dysregulated immune response, decreased availability of NO, increased crosslinking of connective tissue, and fibrosis [39]. Apart from conventional risk factors, changes in heart anatomy and function associated with DM, such as fibrosis, left ventricular (LV) remodeling, and compromised contractility, contribute to the categorization of DCM into four stages or subgroups. They are shown in the Table 1 below [40,41,42].

## 5. Diagnosis

At present, there are no distinct morphological alterations, biochemical indicators, or clinical symptoms that are essential for confirming a diagnosis of DCM. This condition frequently lacks symptoms during its initial phases and frequently coincides with other diabetic complications, complicating the process of making a definitive diagnosis [43]. This condition features a prolonged subclinical period during which significant histological and functional changes may occur, yet clinical symptoms remain absent. Thus, identifying instances within asymptomatic individuals with DM could prove especially beneficial [44]. The most appropriate method for recognizing DCM involves identifying structural and functional alterations in the left ventricle while ruling out other conditions that may contribute to HF. Structural alterations can be detected using both invasive and non-invasive methods, while functional changes are typically identified through echocardiography. Metabolic changes in the diabetic heart can be characterized using techniques such as magnetic resonance spectroscopy, positron emission tomography (PET) and single-photon emission computed tomography [43,44,45]. Specific biomarkers that can aid in diagnosis are still being investigated. In recent years, cardiac microRNAs (miRNAs) have emerged as crucial regulators in the development of DCM. miRNAs found circulating in the blood are highly stable and hold promise as potential diagnostic and prognostic biomarkers. Several miRNAs have been identified in the plasma or whole blood of diabetic individuals. These miRNAs target specific mRNAs that are altered in DCM, playing significant roles in regulating genes associated with the key pathophysiological pathways of DCM, such as hypertrophy, apoptosis, and fibrosis [46,47]. Additional novel parameters associated with cardiac alterations in individuals with DM can be recognized. These encompass inflammatory and metabolic biomarkers linked to mitral annular calcification (MAC). An analysis of insulin resistance, inflammation, and hepatic steatosis markers in patients with T2DM lacking atherosclerotic manifestations but with incidental echocardiographic detection of mild MAC was conducted. While various potential markers were considered, the analysis highlighted TNF-α for inflammation and HOMA C-peptide for insulin resistance as particularly significant. The results show that the generally available routine tests and echocardiographic evaluations are beneficial for the early detection of mitral annular calcifications in diabetic patients [48]. Diagnostic methods for DCM are shown in the Table 2 below [11,48,49].

A meta-analysis explored the potential diagnostic utility of three-dimensional echocardiography with speckle tracking in identifying DCM. This analysis observed a decrease in myocardial strain in asymptomatic individuals with subclinical DM compared to healthy counterparts. Various parameters including global longitudinal, circumferential, radial, and superficial strain were assessed. Results revealed that patients with DM exhibited higher strain values than healthy subjects, with the most significant reduction observed in global longitudinal strain. These findings suggest that three-dimensional tracking echocardiography could be beneficial in diagnosing subclinical DCM, with particular emphasis on the sensitivity of 3D longitudinal strain as a marker for detecting subclinical left ventricular dysfunction in diabetic patients [50].

## 6. Treatment

**Figure 6:**
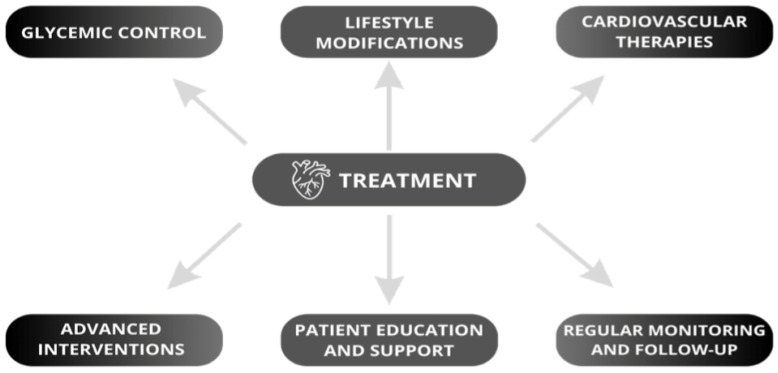
Management approach for diabetic cardiomyopathy: integrating lifestyle, glycemic control, and cardiovascular therapies [51,52].

Managing DCM involves a comprehensive approach that includes lifestyle modifications, glycemic control, and targeted cardiovascular therapies (Figure 6) [51,52].

Lifestyle modifications comprise dietary changes, regular exercise along with quitting smoking. Individuals with DCM should follow a heart-healthy diet that is low in saturated and trans fats, cholesterol, and sodium [53,54]. Monitoring and controlling calorie intake are crucial to prevent obesity, which is a risk factor for both DM and CVD [54,55,56]. Furthermore, physical activity is essential in managing DM and decreases the occurrence of cardiac events, including heart attacks and strokes [57]. Regular exercise helps control blood sugar levels, improve cardiovascular health, and maintain a healthy weight [51,58]. However, individuals should consult with healthcare providers before starting a new exercise regimen [51]. Smoking is a major risk factor for heart disease, and individuals with diabetes who smoke are at an increased risk of developing DCM. Quitting smoking is a crucial step in managing this condition [14,36,59].

It is worth mentioning that together with lifestyle modifications proper glycemic control is crucial. Medications such as insulin, oral hypoglycemic agents, and other hypoglycemic drugs may be prescribed to maintain optimal blood glucose levels. Healthcare providers may adjust medication regimens based on individual patient needs and response to treatment [43,60,61].

Cardiovascular therapies, including blood pressure and lipid management, along with antiplatelet therapy, play a crucial role [62,63,64]. In advanced cases, device therapies and, rarely, heart transplantation may be considered [65]. Regular monitoring through routine check-ups and diagnostic tests, alongside patient education and support, form the cornerstone of effective management. This effort between healthcare providers and patients ensures a comprehensive and multidisciplinary approach, optimizing long-term cardiovascular health and mitigating the impact of DCM [66].

### 6.1. Non-Pharmacological Treatment

**Figure 7:**
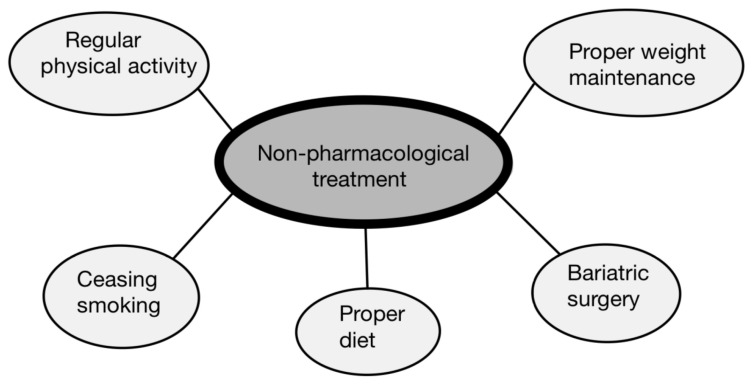
Pillars of non-pharmacological treatment in diabetic cardiomyopathy.

Undoubtedly, there exists a reciprocal relationship between DM and HF, in which each condition exerts an influence on the other one. HF leads to increased activity of both the renin–angiotensin–aldosterone system and the sympathetic nervous system. On the other hand, hyperglycemia induces micro- and macroangiopathic complications, resulting in coronary artery disease, cardiomyocyte fibrosis and sarcomere stiffness. For this reason, non-pharmacological treatment should be directed against both diabetes and HF [67]. Especially important are lifestyle changes such as regular physical activity, maintaining a healthy weight and ceasing smoking [68]. In the context of physical activity, particular attention should be paid to aerobic training, which has the ability to reverse left ventricular remodeling in patients suffering from HF [69]. However, there is also no need to fear interval training, which involves performing short bouts of exercises at relatively high intensity, followed by long rest periods. Similar to the previous form of exercise, this one also has the ability to counteract detrimental remodeling of the cardiac muscle [70]. In addition to its impact on the heart structure, regular physical activity also leads to a reduction in the levels of pro-inflammatory factors in the blood, as well as an increase in anti-inflammatory substances such as IL-10. Moreover, physical activity lowers sympathetic nervous system activity, reduces levels of catecholamines and angiotensin-II in the bloodstream. Furthermore, it exerts a positive influence on endothelial function, mainly through elevated nitric oxide synthase production [71]. In relation to DM, it is particularly noteworthy that various forms of PA have significant potential to reduce hyperglycemia and improve overall glycemic control [72]. Another important aspect limiting the development of DCM is diet. This area requires further research; however, the current emphasis is on reducing the consumption of saturated FA and salt. Equally important is a low-carbohydrate diet, which not only improves glycemic control, but also possesses the capacity to deter detrimental postprandial alterations associated with glucotoxicity and lipotoxicity [73]. Another element of non-pharmacological treatment for DCM is bariatric surgery. Despite its invasiveness, this treatment method offers several advantages, including better control of blood pressure, reduced overall cardiovascular risk and others related to those events. Moreover, in severely obese patients, this procedure improves left ventricular end-diastolic dimension and reduces hospitalization time in the HF patients’ group. This method, due to a notable reduction in adipose tissue, lowers the afterload and preload, as well as improving both local and systemic energy requirements. What is more, bariatric surgery may improve mitochondrial and endothelial functioning [74]. Just as important appears to be smoking cessation, which constitutes an important modifiable risk factor for HF. This is because smoking cigarettes is correlated with heightened arterial rigidity, accelerated heart rate and increased overall mortality due to HF [75]. The last, but not least is a reduction in excess body weight, which, in addition to the possible improvement in cardiac muscle function, also inhibits the development of T2DM or even reverses its negative effects (Figure 7) [51].

### 6.2. Hypoglycemic Drugs

Traditional methods used to treat DCM include: maintaining normal blood glucose [76], health-promoting behaviors, and pharmacotherapy for HF. These therapeutic approaches reduce the risk of developing DCM and minimize the chance of cardiovascular complications [13]. Anatomical and functional abnormalities of the left ventricle, which are not caused by cardiac ischemia, can be a consequence of DM. Adequate treatment of DM, a major cause of DCM, can reduce morbidity and mortality in patients with hyperglycemia [77].

#### 6.2.1. Metformin

For years, one of the preferred drugs used to maintain euglycemia in patients with T2DM has been metformin [76]. It owes its popularity to its high efficacy at a relatively low price and good safety profile [78]. This oral drug, which belongs to the group of biguanide derivatives, has been used in patients suffering from HF since 2006. Previously, it was thought that metformin could lead to lactate acidosis in these patients [79]. However, analyses have shown its safety and reduced mortality in patients struggling with HF as well. The primary mechanism of action of this drug is the inhibition of gluconeogenesis, which results in reduced hepatic glucose production [80]. Its exact mechanism of action is still the subject of much research, as it was not a drug created to act on a targeted metabolic pathway or disease entity, but a substance that is a plant derivative that was used in herbal medicine as early as the Middle Ages [81].

A study using PET showed that metformin accumulates in the liver, intestines and urinary tract from where it is eliminated unchanged [82]. The drug can be safely used in patients with a glomerular filtration rate of more than 30 mL/min/1.73 m^2^ [78]. In the observation of animal models, it was noted that metformin regulated the autophagocytosis of myocardial cells, protecting them from developing DCM [13]. The UKPDS in their study showed a correlation between the level of HbA1c and the risk of death from cardiovascular causes. (As little as a 1% increase in HbA1c increased the possibility of death from CVD by more than 10%) [76]. The effectiveness of metformin in glycemic control depends on the dose taken and the length of therapy [81,82]. A study by Garber’s team showed a 0.9% reduction in HbA1c levels over 14 weeks in a group of patients with T2DM who took the biguanide derivative at a dose of 500 mg. In contrast, a 2% decrease in HbA1c was observed in a group of diabetics taking metformin at four times the dose [82]. The UKPDS study showed that the cardioprotective effect of the biguanide derivative was most likely not only due to adequate blood glucose control, but to the specific effect of metformin in reducing macrovascular complications. The presented conclusions were argued by the fact that other drugs tested in the study (insulin or sulfonylurea derivatives), despite equally good glycemic control, did not induce as good cardiovascular benefits as metformin [78].

#### 6.2.2. Sodium Glucose Cotransporter 2 (SGLT2) Inhibitors

Recent studies have shown improved myocardial contractile function in diabetics using sodium glucose cotransporter 2 (SGLT2) inhibitors and glucagon-like peptide 1 (GLP-1) receptor agonists [4]. The distinguishing feature of SGLT2 inhibitors from other drugs used in the treatment of diabetes is their unique mechanism of eliminating glucose from the body. Unlike most drugs used, SGLT2 inhibitors do not cause excessive insulin secretion, thus protecting patients from dangerous drops in blood glucose levels [79].

Physiologically, approximately 160–180 g of glucose per day is reabsorbed in the glomeruli, resulting in no glucose in the urine of healthy individuals. This changes in type 2 diabetics, in whom the amount of glucose excreted in the urine increases with increasing glycaemia [80]. Nearly 90% of the glucose is reabsorbed in the proximal tubule of the kidney, where SGLT2 is located, while a second form of this cotransporter-SGLT1 is located slightly further downstream in the proximal tubule (approximately 10% reabsorption). It has been noted that in patients with DM, SGLT2 inhibitors inhibit glucose reabsorption in the renal proximal tubule, resulting in an increase in urinary glucose elimination and concomitant control of glycaemia [83].

In 2015, a study involving a group of 7020 T2DM patients tested the effect of empagliflozin on their body compared to a placebo for more than three years [84]. This randomized clinical trial—Empagliflozin Cardiovascular Outcome Event Trial in Type 2 Diabetes Mellitus Patients (EMPA-REG OUTCOME)—was the first to show a positive effect of a representative of the SGLT2 inhibitor group (empagliflozin) on CVD risk reduction [35]. Empagliflozin is distinguished from other SGLT2 inhibitor drugs by its high selectivity for SGLT2 inhibition (it is more than 2500-fold higher compared to SGLT1 [85,86].

The EMPA-REG OUTCOME study showed a surprisingly positive effect in the empagliflozin group on the risk of death from cardiovascular events (almost 40% fewer deaths due to cardiovascular events), a 35% reduction in the need for hospitalization was observed in the HF group and a more than 30% reduction in the risk of death regardless of the type of comorbidities present [35]. Further studies conducted evaluated the effect of canagliflozin and dapagliflozin in reducing cardiovascular events. The Dapagliflozin Effect on Cardiovascular Events–Thrombolysis in Myocardial Infarction Study (DECLARE-TIMI) and the Canagliflozin Cardiovascular Assessment Study (CANVAS) showed that canagliflozin, although not significantly reducing mortality, reduced hospitalization in patients with HF and reduced the adverse events studied, such as stroke and myocardial infarction, which did not lead to loss of life or myocardial infarction, which also did not end in death. Of the agents studied, the second phlorizin (dapagliflozin) had a beneficial effect only by reducing the incidence of hospitalization in the HF group [35,84].

In conclusion, the meta-analyses conducted show a beneficial effect from the tested SGLT2 inhibitors on the reduced incidence of hospitalization in HF patients and the reduced risk of CVD death. In addition, the inhibition of renal disease in study patients was demonstrated [84]. Importantly, although the drug is removed from the body almost equally by the intestines and by the kidneys, only hypoglycemic episodes were seen in patients in stage 4 chronic kidney disease. The safety of the drug is supported by the fact that empagliflozin and other antidiabetic drugs have not been observed to interact, and the few restrictions on the use of empagliflozin include pregnancy and breastfeeding [86].

#### 6.2.3. Insulin

**Figure 8:**
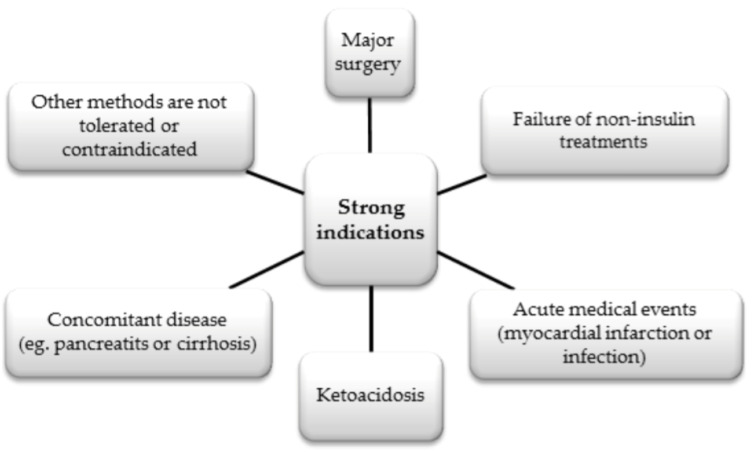
Strong indications for insulin use in DM2 patients for adequate glycemic control [88].

Insulin, which was discovered in 1921, is a mainstay in the treatment of people with T1DM [87]. In some cases in patients with T2DM, there are also strong indications for its use, as shown in Figure 8 below [88].

The functions of exogenous insulin include enhancing the ability to metabolize carbohydrates, transforming glycogen into fat and holding glucose stores in the liver [89].

#### 6.2.4. Dipeptidylpeptidase-4 Inhibitors

The mechanism of dipeptidylpeptidase-4 (DPP-4) inhibitors is a postprandial 2–3-fold increase in plasma GLP-1 by inhibiting DPP-4 contributing to reduced glucagon secretion and insulin stimulation [90]. All GLP-1 inhibitors currently approved for use show efficacy in reducing glycemic levels at a very similar, moderate level (they reduce HbA1c by less than 1%) [91]. A relatively new oral representative of DPP-4 inhibitors is evogliptin, which in 2015 was approved by the Ministry of Food and Drug Safety of Korea for use in people with T2DM to lower glucose levels [83,84]. In order to test the effect of this drug on the development of DCM, a study was conducted in mice, from which the following conclusions were drawn: after 3 months of treatment, no effect on body weight was observed, only a slight decrease in cholesterol or triglycerides (TG) was noted, but a clear decrease in glycaemia and HbA1c was proven [92].

Currently, we do not have sufficient evidence to assess the effect of evogliptin on cardiovascular events, and large-group studies are needed to determine the safety profile of this drug and its efficacy in reducing the cardiovascular side effects of diabetes [93].

#### 6.2.5. Thiazolidinediones

A representative of the thiazolidinediones group, pioglitazone, was also of interest in the studies conducted. It was shown that, in a group of diabetic patients, it increased fluid retention in the body by up to 10% and contributed to weight gain. This translated into a deterioration of cardiac function and an increased risk of hospitalization in patients [76]. As early as 2016, the European Society of Cardiology made it clear in its guidelines that these drugs should not be used in patients with HF. In earlier years, they were only allowed to be used in patients in NYHA class I-II [35]. Rosiglitazone is another drug in this group that has received special attention by being shown to be associated with an increased risk of myocardial infarction [86].

#### 6.2.6. Dual GIP/GLP-1 Receptor Agonist

**Figure 9:**
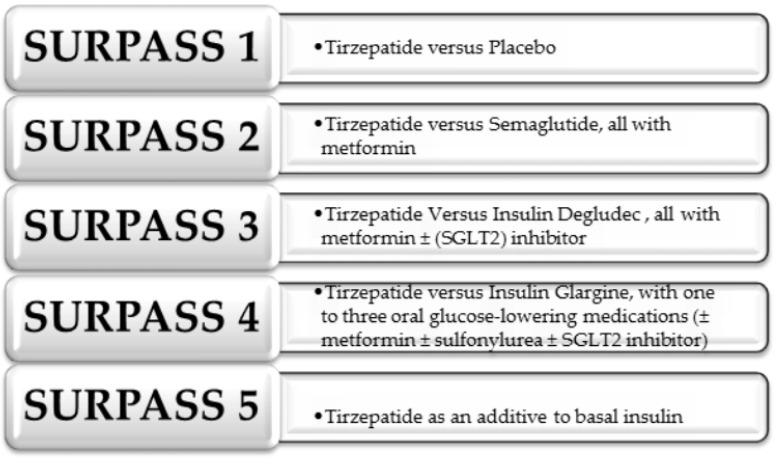
The SURPASS 1-5 study as an evaluation of tirzepatide in monotherapy or in combination with other known hypoglycemic drugs [96,100].

The beginning of 2016 brought a new glycemic control drug proposed by Eli Lilly [94]. It is tirzepatide (LY3298176), a dual GIP/GLP-1 receptor agonist, which has been approved by the U.S. Food and Drug Administration as a drug for the treatment of T2DM [94,95,96]. This hypoglycemic agent is a synthetically produced linear peptide composed of 39 amino acids [95]. Thanks to the addition of the C20 fatty acid diquat molecule, the half-life has been extended to as much as 5 days which translates into the possibility of using this drug subcutaneously only once a week [94,95,97,98]. In order to evaluate the efficacy and safety profile of tirzepatide in patients with T2DM, the SURPASS program was established, including a number of studies including SURPASS 1–6 and the SURPASS CVOT study conducted in Asia to evaluate the effect of the drug on cardiovascular risk (however, we currently do not have access to these data, although the results of this study are expected to be published as early as this year) [99]. The SURPASS 1–5 trials evaluated the efficacy of tirzepatide at doses of 5, 10 and 15 mg in monotherapy or in combination with another known hypoglycemic drug as shown in Figure 9 below [96,100].

In the 40-week randomized, double-blind SURPASS-1 trial, patients were randomly assigned to receive placebo or tirzepatide at doses of 5, 10 or 15 mg [101]. Compared to placebo, each dose of the drug showed a reduction in body weight and HbA1c levels in the subjects [96,99,102]. The SURPASS 1–5 study showed that tirzepatide, at each of the doses tested, was more effective in reducing body weight (from 5.4 to 11.7 kg) and lowering HbA1c than other known hypoglycemic drugs taken by patients during the study [96,99]. Interestingly, it was noted from the study that tirzepatide was effective in reducing body weight even in patients taking drugs associated with weight gain, such as sulfonylurea derivatives or insulin, as their primary therapy [100]. Sustained weight reduction may favorably influence T2DM remission, thereby reducing patients’ cardiovascular risk and contributing to a decrease in mortality [102]. In the SURPASS 1–5 study, a decrease in fasting glucose levels of 2.3 to 3.5 mmol/L was observed. In addition, the drug’s effect on lowering: TG levels (by as much as 24.8%), LDL cholesterol (by as much as 12.4%) systolic blood pressure (SBP) (by 5–6 mmHg) and inflammatory parameters like CRP was noted [99]. Undoubtedly, this new therapy based on the use of dual GIP/GLP-1 agonists having not only hypoglycemic effects, but also exerting weight reduction and regulating lipid disorders has the potential to become a new therapeutic potential in DCM. However, for the time being, more studies are needed to assess the drug’s long-term impact on possible cardiovascular benefits [103].

### 6.3. Lipid-Lowering Drugs-Statins

The dysregulation of lipid metabolism in diabetes significantly contributes to the development and progression of cardiovascular complications. Elevated levels of TG, low-density lipoprotein cholesterol (LDL-C), and decreased high-density lipoprotein cholesterol (HDL-C) create a proatherogenic environment. This dyslipidemia, coupled with insulin resistance, combine into oxidative stress and inflammation within the cardiovascular system, leading to myocardial remodeling, thus potentially exacerbating DCM [104,105,106]. Only just over 25% of people with T2DM accomplish the LDL-C target, attempting to regulate dyslipidemia, one of the worst regulated conditions in the world [107]. 

While diet and lifestyle adjustments remain the foundational components of diabetes-related atherosclerotic CVD prevention, statin medication continues to be the gold standard pharmacological intervention [108]. The alternative medications have been investigated for alleviating cardiac damage because glucose-oriented therapy is inadequate in preventing the cardiac consequences of long-term T2DM [109]. Because of their anti-inflammatory properties, statins have been proposed to have a protective role in the context of DCM. Additionally, the diabetic myocardium’s lipotoxicity and intracardiac FA formation are linked to hyperlipidemia [110]. Statins inhibit the enzyme HMG-CoA reductase, a key player in cholesterol synthesis, leading to a reduction in circulating LDL-C levels [111]. Additionally, apart from LDL-cholesterol reduction, statins exhibit pleiotropic effects, including anti-inflammatory and antioxidant properties, which may confer cardiovascular benefits [112,113,114]. Oesterle et al. suggested that statins may also have possible anti-arrhythmic effect on atrial fibrillation and ventricular tachyarrhythmias [115]. Studies have shown that statins may have potential benefits in alleviating the progression of DCM. By modulating lipid profiles, statins address a key contributor to the development of myocardial fibrosis and dysfunction. Moreover, the anti-inflammatory and antioxidant properties of statins may help counteract the deleterious effects of oxidative stress and inflammation within the diabetic myocardium. However, the exact mechanisms underlying these benefits are complex and multifaceted [116]. Preclinical data show that atorvastatin may enhance left ventricular function in an animal model of DCM by lowering myocardial fibrosis and cardiac intramyocardial inflammation [117]. Further, in DCM, atorvastatin restored the β-adrenoceptor stimulation’s beneficial inotropic effect. Multiple changes in the expression of proteins in the β-adrenergic signaling pathway, particularly through the NOS synthase pathway, facilitate this impact [118]. Additionally, rosuvastatin also showed protective effects by decreasing the NLR family pyrin domain containing 3 (NLRP3) inflammasome and IL-1β activation through the regulation of mitogen-activated protein kinase (MAPK) pathways [119]. Simvastatin might alleviate the effects of hyperglycemia on myocardial oxidative stress, inflammation, and apoptosis in streptozotocin-induced diabetic rats. In the therapeutic setting, people with diabetes who receive rigorous cholesterol control with statins and other medications have a significant reduction in their cardiovascular risk [120,121,122,123]. Therefore, diabetic guidelines recommend statins for both primary and secondary CVD prevention [124].

## 7. The NLRP3 Inflammasome

The nucleotide-binding and oligomerization domain-like receptor family pyrin domain-containing 3 (NLRP3) inflammasome is a multiprotein complex that is activated in response to various stressors, including metabolic dysregulation and oxidative stress, both of which are prominent features of diabetes. Activation of the NLRP3 inflammasome leads to the cleavage and activation of pro-inflammatory cytokines such as IL-1β and IL-18, contributing to chronic low-grade inflammation [125,126]. The NLRP3 inflammasome has been implicated in the pathogenesis of arteriosclerosis, by promoting inflammation, oxidative stress, and vascular cell dysfunction [127]. It is worth mentioning the involvement of the NLRP3 inflammasome in the pathogenesis of DCM. High glucose induces myocardial damage by activating the NLRP3 inflammasome [128]. A dysregulated activation of the NLRP3 inflammasome leads to an overproduction of cytokines [129]. Sun et al. suggests that DCM is an inflammatory disease aggravated by NLRP3 inflammasome-mediated release of IL-1β and IL-18. Therefore, modulating the NLRP3 inflammasome could be a promising therapeutic strategy for the treatment of DCM [130]. Moreover, the release of pro-inflammatory cytokines by the NLRP3 inflammasome can exacerbate inflammation and fibrosis in the heart tissue, contributing to the progression of DCM [29,131]. Targeting the NLRP3 inflammasome pathway may represent a promising therapeutic strategy for the treatment of DCM by alleviating cardiac inflammation and dysfunction. However, further research is needed to fully elucidate the underlying mechanisms and validate the therapeutic potential of targeting the NLRP3 inflammasome in DCM [132].

## 8. Complications, Prognosis and Novel Therapeutic Options

DCM entails certain functional and structural complications, including among others fibrosis, myocardial hypertrophy, increased rigidity and loss of cardiomyocytes. This results from a disrupted glucose and lipid metabolism, which impacts the interaction between excitation, contraction and initiates oxidative stress and AGEs creation. This also disturbs the usual control of cardiomyocyte proliferation and enlargement. Currently, therapeutic approaches for cardiovascular complications linked to diabetes depend on treatment aimed at glycemia control, decreasing lipid concentration and mitigating oxidative stress; however, novel treatment methods are being sought [133]. Thus far, endeavors have been undertaken, for example, to employ phosphodiesterase type 5A inhibitors, revealing that prolonged inhibition of this enzyme exhibits an anti-remodeling impact on the left ventricle and enhances cardiac muscle kinetics [134]. An especially intriguing direction in potential novel DCM therapy involves the utilization of cardiac stem cells. These cells have the capacity to differentiate into cardiomyocytes, a population that diminishes continually in DCM. What is more, equally noteworthy is the therapeutic avenue where miRNAs could be employed. Altering miRNA expression has shown the ability to reverse the functional and histological indicators of DCM in animals. Furthermore, consideration is given to the application of visceral adipose tissue-derived serine protease inhibitor, phosphoinositide3-kinase gamma inhibitor, antioxidants, or coenzyme Q10 in DCM treatment, but further research on their effectiveness needs to be conducted [18].

## 9. Conclusions

In conclusion, early recognition and management of DCM are crucial for individuals with DM. Epidemiological studies highlight the strong link between DM and HF, emphasizing the need for proactive intervention. Risk factors such as prolonged hyperglycemia contribute to DCM development. Diagnosis can be challenging due to the asymptomatic nature of DCM in its early stages, but advanced imaging techniques and biomarkers offer valuable tools for detection. Continued research is essential for improving our understanding of DCM and enhancing patient outcomes in diabetic individuals. In the context of DCM, both HF and DM are interrelated and mutually influence each other. As a result, the focus should be on treating both of these mentioned disease entities. In addition to pharmacological treatment, regular physical activity plays a crucial supportive role in therapy, improving not only cardiac function but also glycemic levels. It is also important to pay attention to the nutrition of patients suffering from DCM and introduce a low-carbohydrate diet, as well as encourage weight reduction, including bariatric surgery. At present, DCM treatment mainly revolves around controlling blood glucose levels, reducing oxidative stress, and lowering blood lipid levels. However, there are increasingly noteworthy novel methods of treatment emerging, such as the utilization of cardiac stem cells or miRNAs. In conclusion, the management of DCM involves a multifaceted approach that addresses lifestyle factors, glycemic control, and targeted cardiovascular therapies. A collaborative effort between healthcare providers and patients is crucial for developing individualized treatment plans and achieving optimal outcomes. Regular monitoring, adherence to medications, and lifestyle modifications are key components of managing DCM and preventing its progression to advanced stages. One of the primary treatments for DCM includes proper glycemic control. We can achieve it using both relatively new drugs like the phlazines or the still-investigational DPP4 inhibitors, but the long-known diabetes drugs like insulin or metformin will work just as well here. Studies have shown that TZDs should be avoided in this group of patients, which, among other things, worsen cardiac function and increase the risk of hospitalization through increased fluid retention.

In individuals with diabetes, there is an increased risk of HF and statins have been explored for their cardiovascular benefits beyond lipid-lowering effects. Studies suggest that statins, such as atorvastatin, rosuvastatin, and simvastatin, exhibit protective properties by reducing cardiac inflammation, fibrosis, and dysfunction associated with DCM. In particular, by reducing lipids and inflammation, statins can influence the pathogenesis of both myocardial infarction and DCM. A promising approach to improving outcomes and resolving the complex issues highlighted by DCM is the integration of lipid-lowering medications into customized therapy regimens. Therefore, further research is needed to better understand how they specifically affect DCM. Several studies have demonstrated the involvement of the NLRP3 inflammasome in the pathogenesis of DCM. What is worth mentioning, the development of inhibitors targeting specific aspects of inflammation suggests that NLRP3 inflammasome can be used to treat DCM.

## Figures and Tables

**Table 1:** Stages/subgroups of DCM [40,41,42].

	Stage 1	Stage 2	Stage 3	Stage 4
**Advancement of phase**	Early phase	Middle phase	Middle/late phase	Late phase
**Type of dysfunction**	Diastolic dysfunction	Diastolic and systolic dysfunction	Diastolic and systolic dysfunction	Diastolic and systolic dysfunction
**Anatomical changes**	Hypertrophy, increased LV mass	Hypertrophy, increased LV mass and wall thickness, fibrosis, dilatation	Fibrosis, dilatation, microangiopathy	Fibrosis, dilatation, microangiopathy and macroangiopathy
**Troponins**	Not elevated	Not elevated	Elevated during inflammation/ischemia	Elevated during infarction or severe HF
**HF symptoms in NYHA scale**	NYHA I	NYHA II	NYHA II–III	NYHA II–IV

LV, left ventricular; HF, heart failure; NYHA, New York Heart Association.

**Table 2:** Diagnostic methods for DCM [11,48,49].

Method	Evaluation	Evaluated Criterion
**Echocardiography**	Functional	Mitral inflow for diastolic function
		Tissue Doppler imaging for diastolic and systolic function
	Structural	In two-dimensional echocardiography LV hypertrophy
**Cardiac PET**	Metabolic and hemodynamic	Myocardial metabolic abnormality and disordered blood flow
**Cardiac MRI**	Functional	Late gadolium-enhancement for diastolic and systolic function
	Structural	Myocardial steatosis, LV hypertrophy
	Metabolic	Magnetic resonance spectroscopy for myocardial TG content and PCr/ATP
**Coronary angiography**	Functional and hemodynamic	Mean PCWP and LVEDP for diastolic function, microvascular coronary artery disease
**Serology**	Functional	mi-RNA for contractile function
		BNP for diastolic and systolic function
		Troponin for LV dysfunction
	Structural	MMPs and TIMPs for myocardial fibrosis
**MAC biomarkers**	Inflammatory and metabolic	Increase in TNF-alpha and HOMA-C peptide levels

LV, left ventricular; PET, positron emission tomography; MRI, magnetic resonance imaging; TG, triglyceride; PCr, phosphocreatine; ATP, adenosine triphosphate; PCWP, pulmonary capillary wedge pressure; LVEDP, left ventricular end-diastolic pressure; mi-RNA, micro-ribonucleic acid; BNP, brain natriuretic peptide; MMP, matrix metalloproteinase; TIMP, tissue inhibitor of MMP; MAC, mitral annular calcification.
